# Fate mapping melanoma persister cells through regression and into recurrent disease in adult zebrafish

**DOI:** 10.1242/dmm.049566

**Published:** 2022-09-16

**Authors:** Jana Travnickova, Sarah Muise, Sonia Wojciechowska, Alessandro Brombin, Zhiqiang Zeng, Adelaide I. J. Young, Cameron Wyatt, E. Elizabeth Patton

**Affiliations:** 1 MRC Human Genetics Unit, Institute of Genetics and Cancer, The University of Edinburgh, Western General Hospital Campus, EH4 2XU Edinburgh, UK; 2 CRUK Scotland Centre, Institute of Genetics and Cancer, The University of Edinburgh, Crewe Road South, EH4 2XU, Edinburgh, UK

**Keywords:** Zebrafish, Lineage tracing, Fate mapping, Melanoma, Persister cells, Recurrent disease

## Abstract

Melanoma heterogeneity and plasticity underlie therapy resistance. Some tumour cells possess innate resistance, while others reprogramme during drug exposure and survive to form persister cells, a source of potential cancer cells for recurrent disease. Tracing individual melanoma cell populations through tumour regression and into recurrent disease remains largely unexplored, in part, because complex animal models are required for live imaging of cell populations over time. Here, we applied tamoxifen-inducible *cre^ERt2^/loxP* lineage tracing to a zebrafish model of MITF-dependent melanoma regression and recurrence to image and trace cell populations *in vivo* through disease stages. Using this strategy, we show that melanoma persister cells at the minimal residual disease site originate from the primary tumour. Next, we fate mapped rare MITF-independent persister cells and demonstrate that these cells directly contribute to progressive disease. Multiplex immunohistochemistry confirmed that MITF-independent persister cells give rise to Mitfa^+^ cells in recurrent disease. Taken together, our work reveals a direct contribution of persister cell populations to recurrent disease, and provides a resource for lineage-tracing methodology in adult zebrafish cancer models.

## Abstract (precis)

**Summary:** We fate map melanoma cells from the primary tumour into a persister cell state and show that persister cells directly contribute to recurrent disease.

## INTRODUCTION

Melanoma, a deadly cancer of pigment producing melanocytes, ranks amongst the highest for genetic and transcriptional heterogeneity (Rambow et al., 2019; Travnickova and Patton, 2021). Therapy resistance remains a major challenge for patients with melanoma, with partial or short-term responses characteristic of targeted therapy, eventually resulting in tumour relapse (Patton et al., 2021; Shen et al., 2020b). This resistance can be intrinsic, in which pre-existing primary tumour cell states directly confer resistance, or acquired, whereby melanoma cells develop resistance upon drug exposure either by acquiring new genetic mutations or adapting their transcriptional state (Marin-Bejar et al., 2021; Marine et al., 2020; Shen et al., 2020b). These resistance mechanisms likely occur concurrently, and contribute to the high heterogeneity within melanoma and the persister cell states at the minimal residual disease (MRD) site.

Recently, using single-cell RNA sequencing, we and others have demonstrated high transcriptional heterogeneity and distinct cell states in the primary tumour and MRD, including states with low-to-no expression of pigmentation lineage markers such as melanocyte inducing transcription factor (MITF)-independent populations (Baron et al., 2020; Ennen et al., 2015; Gerber et al., 2017; Rambow et al., 2018; Tirosh et al., 2016; Travnickova et al., 2019). These studies have identified multiple melanoma cell states that have been proposed to drive tumour recurrence, while others are of unknown contribution to disease progression (Travnickova and Patton, 2021). While revealing new concepts about melanoma transcriptional cell states, these studies reflect only a single or few time points, and are thereby limited in what can be understood about the behaviour of individual cell populations over time.

This limitation is particularly critical in the context of cell plasticity in cancer models. Advances in imaging technologies coupled with lineage-tracing methods in zebrafish models now enable the fate mapping of cell populations over time (Mosimann et al., 2011; Pan et al., 2013). The power of an inducible lineage-tracing system is illustrated by advances in understanding of organ development and tissue homeostasis in zebrafish (Carney and Mosimann, 2018; Thunemann et al., 2017). For example, lineage-tracing experiments using the general neural crest marker *sox10* or the recently established melanocyte stem cell marker *tfap2b* provided proof of the existence of multipotent melanocyte precursors during early embryonic development and their contribution to adult zebrafish pigment cell patterning (Brombin et al., 2022; Singh et al., 2016). Similarly, a conditional *cre/loxP* recombination system demonstrated the hierarchy of neuroepithelial progenitors and the functional heterogeneity of neural stem cells in the vertebrate adult brain using neural lineage-specific markers (Galant et al., 2016; Than-Trong et al., 2020). Furthermore, multispectral lineage tracing revealed the mechanism behind myotome generation (Nguyen et al., 2017). The regenerative capacities of zebrafish combined with an inducible lineage-tracing system have been instrumental in understanding the cell origin and lineage restrictions in regenerated organs such as lesioned heart, fins or spinal cord (Briona et al., 2015; Jopling et al., 2010; Tornini et al., 2017). Recently, multicolour tracing has been combined with mosaic mutagenesis using CRISPR-Cas9 in a novel technique called tissue editing with inducible stem cell tagging via recombination (TWISTR) to show that the fitness of mutant clones is controlled by resistance to inflammation (Avagyan et al., 2021). Despite these advances, the application of tamoxifen to control the temporal and spatial activation of Cre (Cre^ERt2^) in adult zebrafish cancer models has been hampered by technical challenges, such as lack of established protocols and tamoxifen toxicity.

Here, we adapt and optimise the inducible *ubi:Switch* lineage-tracing system (Mosimann et al., 2011) for use in an adult zebrafish cancer model to fate map cells through melanoma growth, regression and recurrence. For the first time, we directly capture melanoma cell switching from MITF-independent persister cells to Mitfa^+^ cells to contribute to recurrent disease *in vivo*. Our work supports the concept that targeting persister cells will be critical to delay or prevent recurrent disease.

## RESULTS

### Conditional tamoxifen-induced fluorophore switch in adult zebrafish melanoma

We have previously developed a conditional MITF-dependent zebrafish melanoma model [*Tg(mitfa:BRAF^V600E^);mitfa^vc7^;tp53^M214K^*], in which melanoma regresses and recurs concurrently with the changes in MITF activity controlled by temperature (Travnickova et al., 2019). In this model, MITF activity is controlled by a temperature-sensitive splicing mutation in the *mitfa* gene (*mitfa^vc7^*; zebrafish orthologue *mitfa* is expressed in the body melanocytes) (Johnson et al., 2011; Zeng et al., 2015). At the lower permissive temperature, MITF activity is on and promotes tumourigenesis, while at the higher temperature *mitfa* RNA is still expressed but is not spliced correctly, and thereby MITF protein levels and activity are abolished. Turning off MITF activity results in tumour regression with remaining persister cells at the MRD site (Travnickova et al., 2019).

**Fig. 1.**
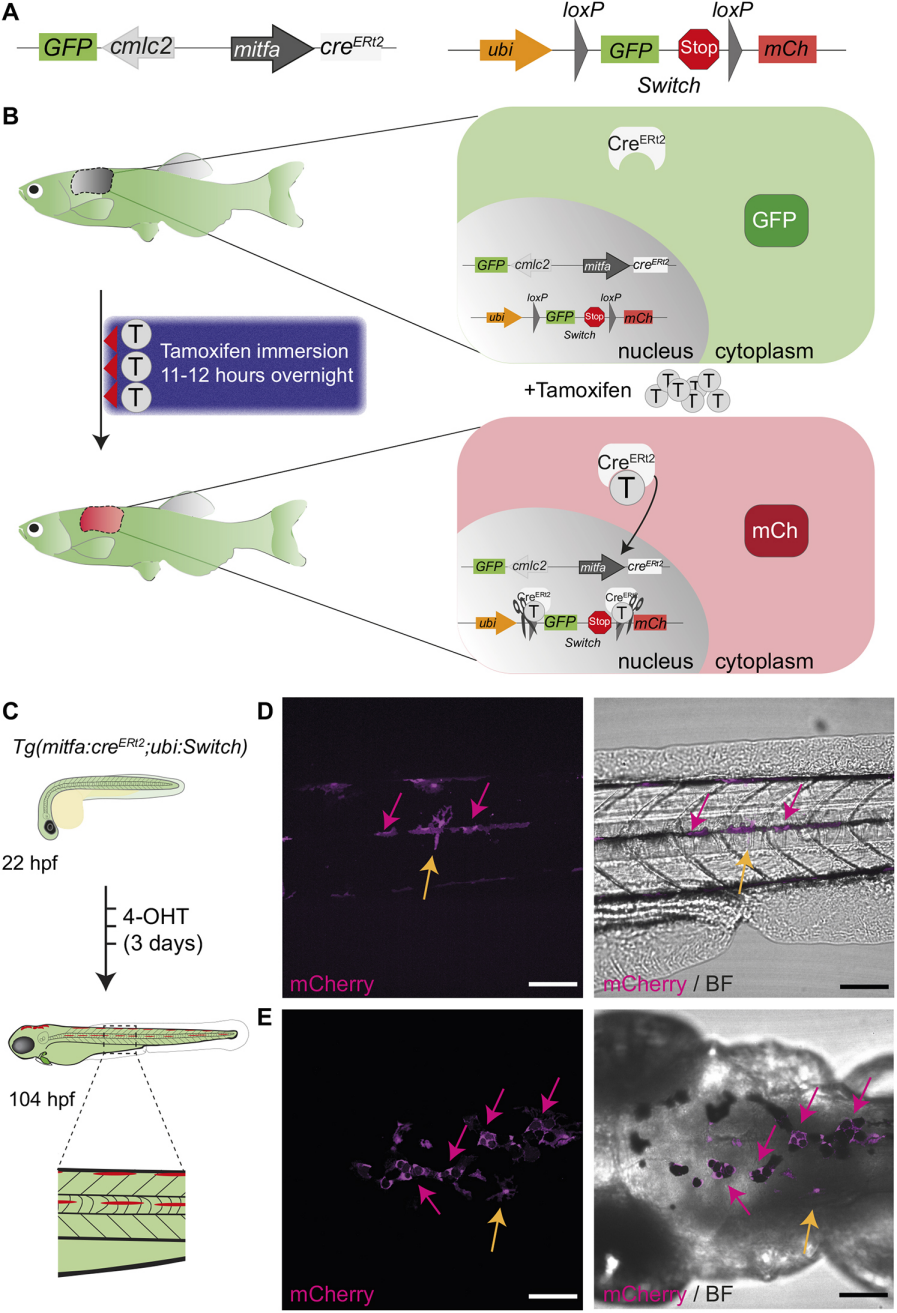
**A tamoxifen-inducible *mitfa:cre^ERt2^/loxP* system in zebrafish labels melanocytes.** (A) A schematic of *mitfa:cre^ERt2^* (left) and *ubi:Switch* (right) (Mosimann et al., 2011) constructs to enable a green-to-red switch in *mitfa* promoter-expressing cells after tamoxifen treatment. The *cardiac myosin light chain 2* (*cmlc2*; also referred to as *myl7*) promoter drives *GFP* expression and facilitates screening for the *cre^ERt2^* line based on GFP^+^ heart myocardium. mCh, mCherry. (B) A schematic of the concept behind the colour-switching *cre^ERt2^/loxP* system that allows green-to-red switch after tamoxifen (T) treatment in cells expressing the *mitfa* promoter driving *cre^ERt2^*. (Left) A three-night course of treatment with tamoxifen for 11-12 h by immersion in the dark with a drug-free period during the day. (Right) The principle of colour switching upon tamoxifen treatment as a result of Cre^ERt2^ trans-localisation to the nucleus, causing excision of *GFP* and a stop codon, enabling mCherry expression in recombined cells. (C) An illustration of the expected green-to-red fluorophore switch in the zebrafish embryo upon three consecutive daily treatments with 20 µM 4-hydroxytamoxifen (4-OHT). Enlarged view of dashed line rectangle shows ‘switched’ melanocytes in the embryonic stripes. hpf, h post fertilisation. (D,E) Lateral (D) and dorsal (E) views of a zebrafish larva after 4-OHT treatment at 104 hpf (4.5 dpf) validates *mitfa:cre^ERt2^* specificity to melanocyte lineage. Standard deviation intensity (STD) projection of a confocal *z*-stack (left, mCherry) and merged with a single *z*-plane acquisition in brightfield (BF) channel (right, merge) at 6 h after the end of 4-OHT treatment. mCherry-expressing melanocytes are visible and express black melanin pigment (pink arrows). Two unpigmented, star shaped cells expressing mCherry are also visible and may represent xanthophore progenitors (yellow arrows). *N*=6 fish for each view, scale bars: 100 µm.

To trace melanoma cells through disease states, we first set out to establish an inducible *cre/loxP* system on the MITF-dependent melanoma background. To this end, we expressed *cre^ERt2^* from the *mitfa* promoter to generate a transgenic *Tg(mitfa:cre^ERt2^)* line and crossed this with a *ubi:Switch* reporter line (Mosimann et al., 2011). This experimental design would enable us to induce a green-to-red (GFP-to-mCherry) permanent fluorophore switch in *mitfa:cre^ERt2^*-expressing melanoma cell populations at a defined time point in melanoma disease progression (Fig. 1A,B).

To validate the specificity of the *mitfa:cre^ERt2^* construct for *mitfa*-expressing cells, we first performed the fluorophore switch during early embryonic development using 4-hydroxytamoxifen (4-OHT) treatment (Fig. 1C). During embryogenesis and in regeneration, Mitfa is required for the generation of melanocytes, and *mitfa* expression marks melanoblasts and progenitors of additional pigment cells, including the yellow xanthophores (Brombin et al., 2022; Parichy et al., 2000). Upon 4-OHT treatment (three consecutive daily treatments of 20 µM), we could detect mCherry-expressing cells at 4.5 days post fertilisation (dpf) along the lateral stipe and on top of the head, in both cases colocalised with pigment (Fig. 1D,E, pink arrows). Some of the mCherry^+^ cells (Fig. 1D,E, yellow arrows) that did not colocalise with pigmented cells likely correspond to pigment cell progenitors (Brombin et al., 2022; Parichy et al., 2000). This indicates that *mitfa:cre^ERt2^* construct specifically labels *mitfa*-expressing cells.

**Fig. 2.**
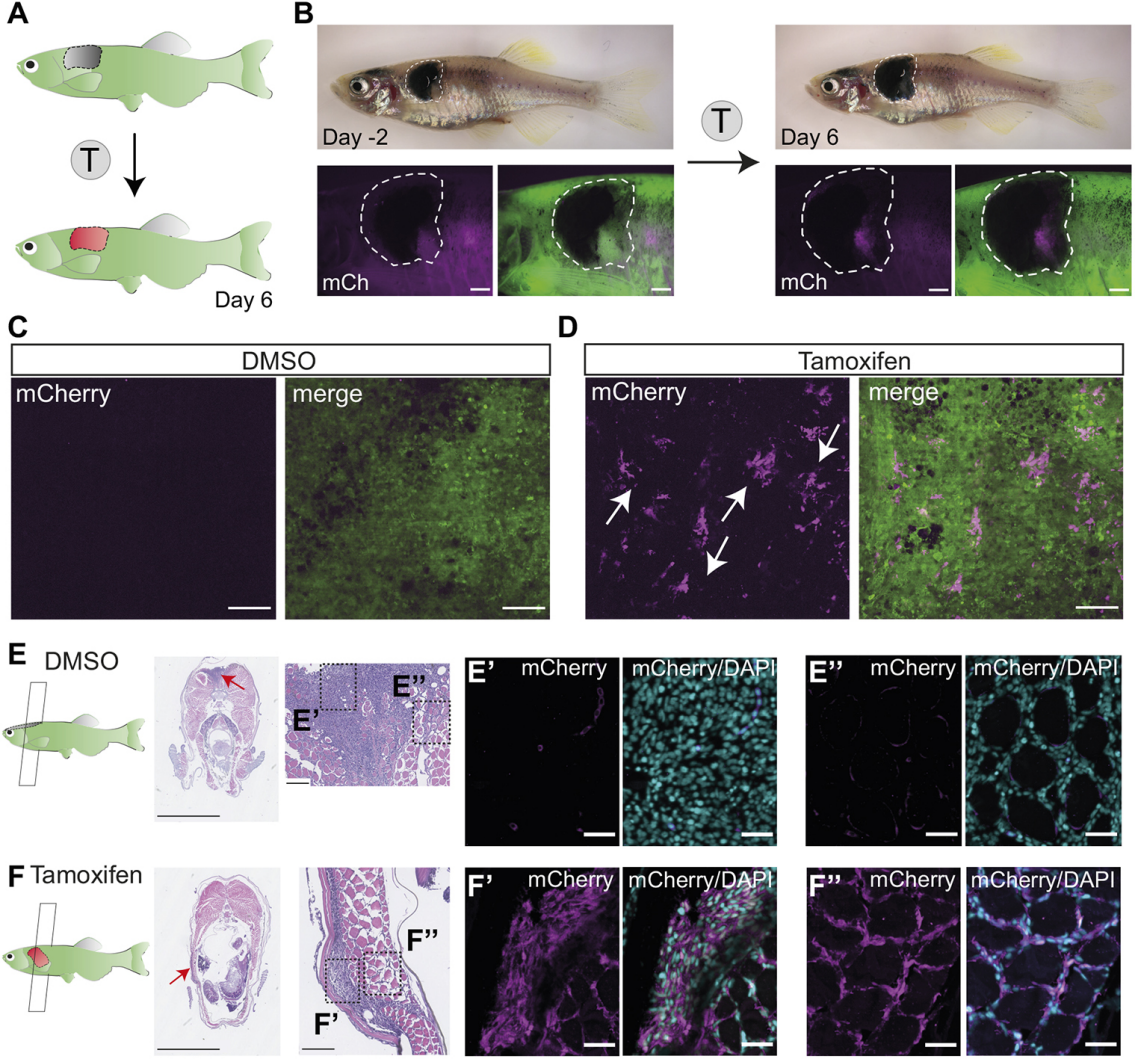
**Successful fluorophore switch in adult zebrafish primary melanoma after tamoxifen treatment.** (A) An illustration of the expected fluorophore switch in the primary melanoma tumour upon tamoxifen (T) treatment and imaged 6 days after the start of the treatment. (B) An adult zebrafish with a fully developed primary melanoma tumour (delineated with white dashed line) 2 days before the tamoxifen treatment course (left) and 6 days after the start of the treatment (right) showing *de novo* expression of mCherry protein in pigmented trunk tumour after tamoxifen treatment (4 µM). *N*=2 fish, scale bars: 1 mm. (C,D) STD projections of confocal *z*-stacks of DMSO (0.04%)-treated fish (C, left mCherry, right merged with GFP) and tamoxifen-treated fish (D, left mCherry, right merge) show small clusters of mCherry-expressing cells in the tamoxifen-treated group only (white arrows). *N*=2 fish, scale bars: 100 µm. (E,F) Paraformaldehyde (PFA)-fixed paraffin-embedded (FFPE) transverse sections of primary melanoma fish (left) stained with Haematoxylin and Eosin, with zoomed images of the tumour (red arrows pointing to the location of the zoomed images). *N*=2 fish, scale bars: 2.5 mm and 100 µm, respectively. Dashed line boxes indicate area of section shown in E′,F′ and E″,F″. (E′,F′) Immunofluorescence staining of bulk tumour with mCherry antibody (magenta), counterstained with the nuclear marker DAPI (cyan), shows mCherry signal in tamoxifen-treated samples only (F′) compared to DMSO control (E′). *N*=2 fish, scale bars: 25 µm. (E″,F″) Immunofluorescence staining of invasive tumour shows successful fluorophore switching and mCherry expression in the tamoxifen-treated group only (F″) compared to DMSO control (E″). *N*=2 fish, scale bars: 25 µm. See also Fig. S1A.

We found that 4 µM tamoxifen treatment of adult zebrafish by immersion for three consecutive nights (11 h treatment, 13 h recovery) was successful for *cre^ERt2^/loxP* recombination without toxicity (Fig. 1B, Fig. 2A). We chose tamoxifen over 4-OHT for its increased solubility in dimethylsulfoxide (DMSO), which is well tolerated by adult zebrafish. The treatment was completed overnight to align with the natural light–dark cycle of the fish and to prevent phototoxicity of tamoxifen (Wang et al., 2009). By 3 days post tamoxifen treatment course, we could detect mCherry in the primary tumour, but not in DMSO-treated controls (Fig. 2B; Fig. S1A). Using confocal microscopy, we validated the presence of individual clusters of mCherry^+^ melanoma cells in tamoxifen-treated tumours only (Fig. 2C,D). Immunostaining of melanoma sections with an mCherry antibody showed that mCherry^+^ cells were present both at the surface of the tumour and in the invading melanoma cells along muscle fibres, thus confirming the efficiency of this protocol to fate map cells throughout the body of the melanoma tumour (Fig. 2E,F).

**Fig. 3.**
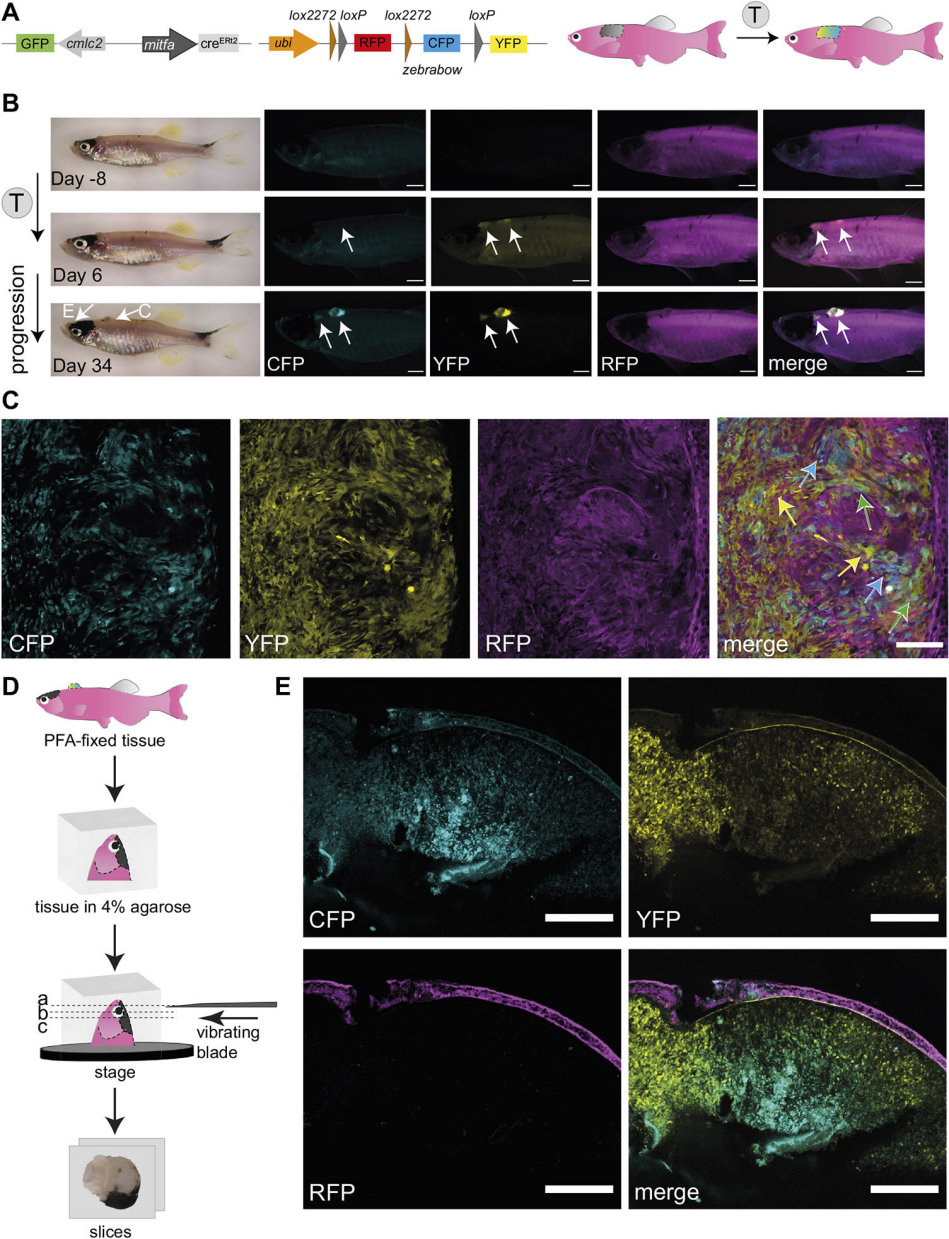
**Successful fluorophore switch using the *zebrabow* system in adult zebrafish primary melanoma following tamoxifen treatment.** (A) A schematic of *mitfa:cre^ERt2^* and *ubi:zebrabow* constructs (Pan et al., 2013), which enables recombination from red signal (RFP) to a stochastic combination of cyan (CFP), yellow (YFP) and red (RFP) signal in *mitfa:cre^ERt2^*-expressing cells after tamoxifen (T) treatment. The *cmlc2* promoter drives *GFP* expression and facilitates screening for the *cre^ERt2^* line based on GFP^+^ heart myocardium. (B) Multicolour labelling in adult zebrafish melanomas. An adult zebrafish with two primary melanomas 8 days before the tamoxifen treatment course (top), and 6 days (middle) and 34 days (bottom) after the start of the treatment. Increasing *de novo* expression of CFP and YFP proteins in both pigmented and unpigmented tumours can be detected after tamoxifen treatments (4 µM). *N*=3 fish, scale bars: 1 mm, white arrows point to two tumour locations. The arrows on the brightfield image (Day 34) point to the melanomas shown in panels C and E, respectively. (C) Confocal multicolour imaging of zebrafish melanoma. Single *z*-plane of confocal acquisition of tamoxifen-treated fish shows individual cells acquiring varied combinations of CFP, YFP and RFP (blue, green and yellow arrows on merged image, respectively). *N*=3 fish, scale bar: 100 µm. (D) Overview of the vibratome sectioning protocol. The PFA-fixed tissue is mounted in 4% agarose and cut using a vibrating blade into 400 µm-thick sections to capture the pigmented tumour (E). (E) Vibratome section imaging of a tamoxifen-treated fish (tissue location as shown in B). Clusters of CFP, YFP or double-expressing cells are clearly visible in areas of the pigmented tumour. STD projections of confocal *z*-stacks of a PFA-fixed sectioned tissue. *N*=3 fish, scale bars: 200 µm. See also Fig. S1B-D.

Having established the system in a single colour switch reporter line, we wanted to test if our method was applicable to the multicolour system of fate mapping. To this end, we crossed the *Tg(mitfa:cre^ERt2^)* melanoma-prone fish with the *ubi:zebrabow* transgenic line (Pan et al., 2013) to evaluate permanent colour changes in *cre^ERt2^*-expressing cells from default red (RFP) to a stochastic combinatorial expression of three fluorophores upon tamoxifen treatment: cyan (CFP), yellow (YFP) and red (RFP) (Fig. 3A). Indeed, similar to *ubi:Switch* line, we could detect *de novo* fluorescent signal by 3 days post tamoxifen treatment compared to DMSO controls, mainly in the YFP channel, and at 34 days post treatment, we could detect all three fluorophores within the primary melanoma (Fig. 3B; Fig. S1B). Using confocal microscopy, we validated the presence of multicolour labelling (CFP, YFP and RFP), which allows distinction of individual cells across the labelled tissue (Fig. 3C; Fig. S1C).

Melanoma tissue often varies in the pigmentation level between individual tumours, and while non-pigmented tumours permit direct detection by fluorescence, intense pigmentation can obscure the fluorescent signal. To overcome this challenge, we performed vibratome sectioning of paraformaldehyde (PFA)-fixed melanoma tissue (Fig. 3D) followed by confocal microscopy. These imaging data show that combining thick tissue sectioning with confocal microscopy permits detection of CFP and YFP channels even in highly pigmented tissues (Fig. 3E; Fig. S1D). While we did not evaluate clonal evolution in melanoma progression (as it would require more rigorous analysis of colour switch stochasticity), this experiment demonstrated the potential for our tamoxifen treatment protocol for application in clonal analysis studies in zebrafish cancer models.

### Melanoma persister cells originate from the primary tumour

**Fig. 4.**
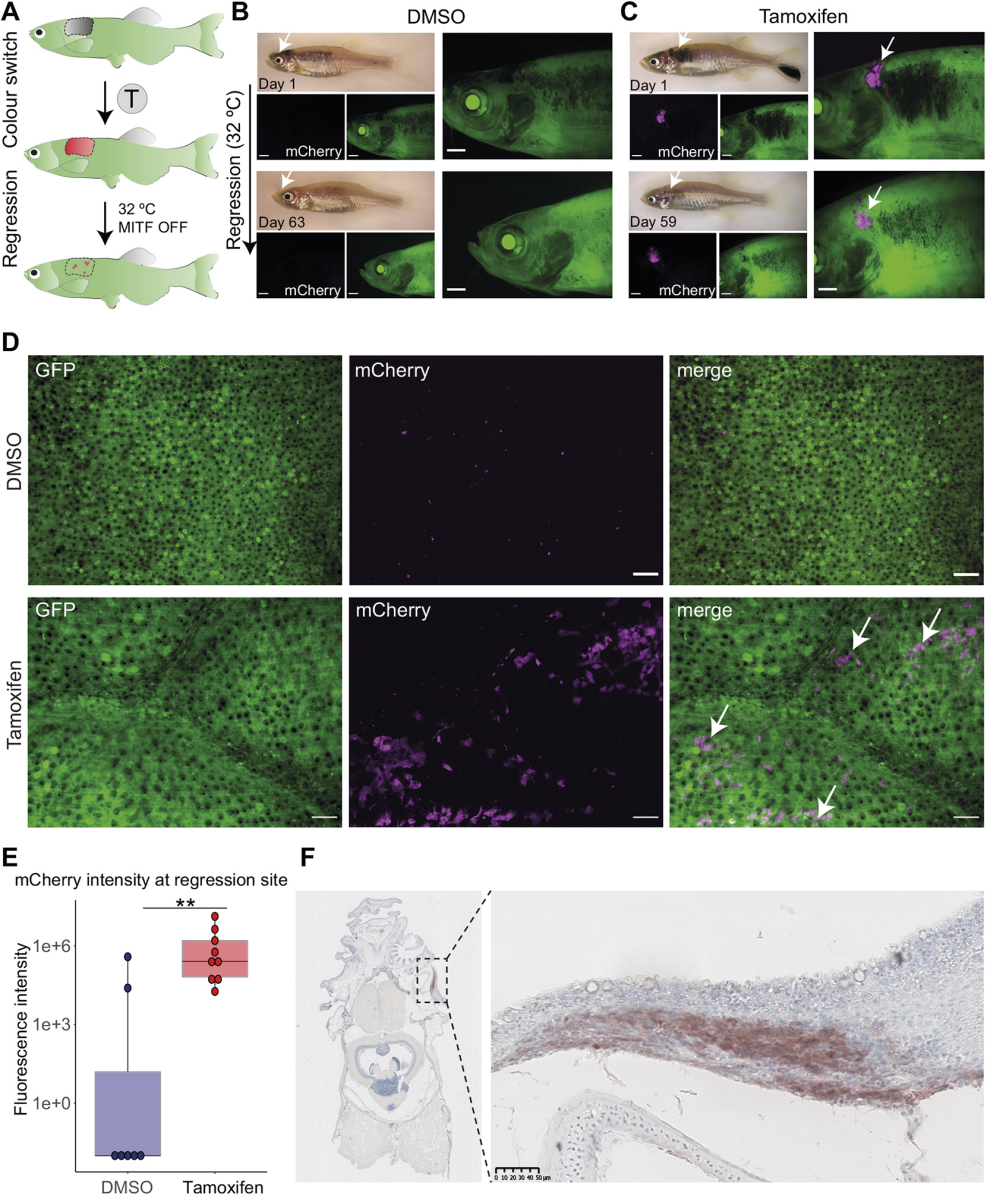
**Persister cells originate from the primary tumour.** (A) The experimental workflow of tamoxifen (T) treatment and melanoma regression at the higher water temperature (32°C, MITF OFF). At the higher temperature, *mitfa* RNA is still expressed but is not spliced correctly and therefore MITF activity is abolished. Loss of MITF activity leads to tumour regression with remaining persister cells at the minimal residual disease (MRD) site. (B) A fish treated with vehicle (0.05% DMSO) showing no green-to-red recombination in the primary tumour (top) or after regression (bottom). Scale bars: 1 mm, white arrows point at the tumour site, *N*=4 fish. (C) A fish treated with tamoxifen (5 µM) showing expression of mCherry^+^ cells in a primary tumour (magenta, white arrows) that are still detected 59 days after initiation of melanoma regression (bottom, white arrows). Scale bars: 1 mm, *N*=7 fish. (D) STD projections of confocal *z*-stack acquisitions of regression sites of fish treated with DMSO or tamoxifen. White arrows point to mCherry^+^ cells (magenta) only present in the tamoxifen-treated condition. Scale bars: 50 μm; DMSO, *N*=4 fish; tamoxifen, *N*=7 fish. (E) Box plot of fluorescence intensity quantification of mCherry signal using average intensity projections of confocal images of residual disease. Lines in boxes indicate the medians, hinges correspond to 25th and 75th percentiles, and whiskers indicate data within 1.5 interquartile range of the upper and lower quartiles. DMSO, *N*=4 fish with seven regression sites; tamoxifen, *N*=7 fish with nine regression sites; **P<0.01, Wilcoxon test. (F) Immunohistochemistry of the MRD site. FFPE transverse section stained with anti-mCherry antibody reveals ‘switched’ cells at the MRD site. Right panel shows an enlarged view of MRD, outlined by the black dashed line rectangle (left panel). Signal in red (AEC substrate) counterstained with nuclear marker Haematoxylin (blue). Scale bar: 50 µm, *N*=3 fish.

Next, we used our conditional MITF-dependent BRAF^V600E^ p53^M214K^ *ubi:Switch* model to trace melanoma cells from the primary tumour as it regresses and thereby determine if the cells detected at the MRD site originate from the primary tumour (Fig. 4A). Tamoxifen treatment of early-stage melanoma resulted in a GFP-to-mCherry switch in tumour lesions that was absent in DMSO-treated animals (Fig. 4B,C). Following a period of tumour growth, we transferred fish to a higher temperature to turn off MITF activity and cause tumour regression until no melanoma was detectable morphologically (5-10 weeks; see Materials and Methods). Strikingly, at the MRD site, we could detect mCherry^+^ persister cells in tamoxifen-treated fish (Fig. 4B,C). Confocal microscopy and quantification confirmed that tamoxifen-treated fish showed significantly greater mCherry signal at the MRD site compared to DMSO-treated fish (Fig. 4D,E). Whilst most DMSO-treated fish did not show any mCherry signal, we could detect it in a small proportion of the control fish (Fig. 4E). This may be due to *cre* expression (leakage) in those tissues where *mitfa* expression is very high [for example in some nodular tumours, as we have described previously (Travnickova et al., 2019)]. Immunostaining of regressed melanoma tissue sections confirmed the presence of mCherry^+^ persister cells at the MRD site (Fig. 4F). These data indicate that primary melanoma cells directly give rise to persister cells at the MRD site.

### Melanoma persister cells directly contribute to tumour recurrence

**Fig. 5.**
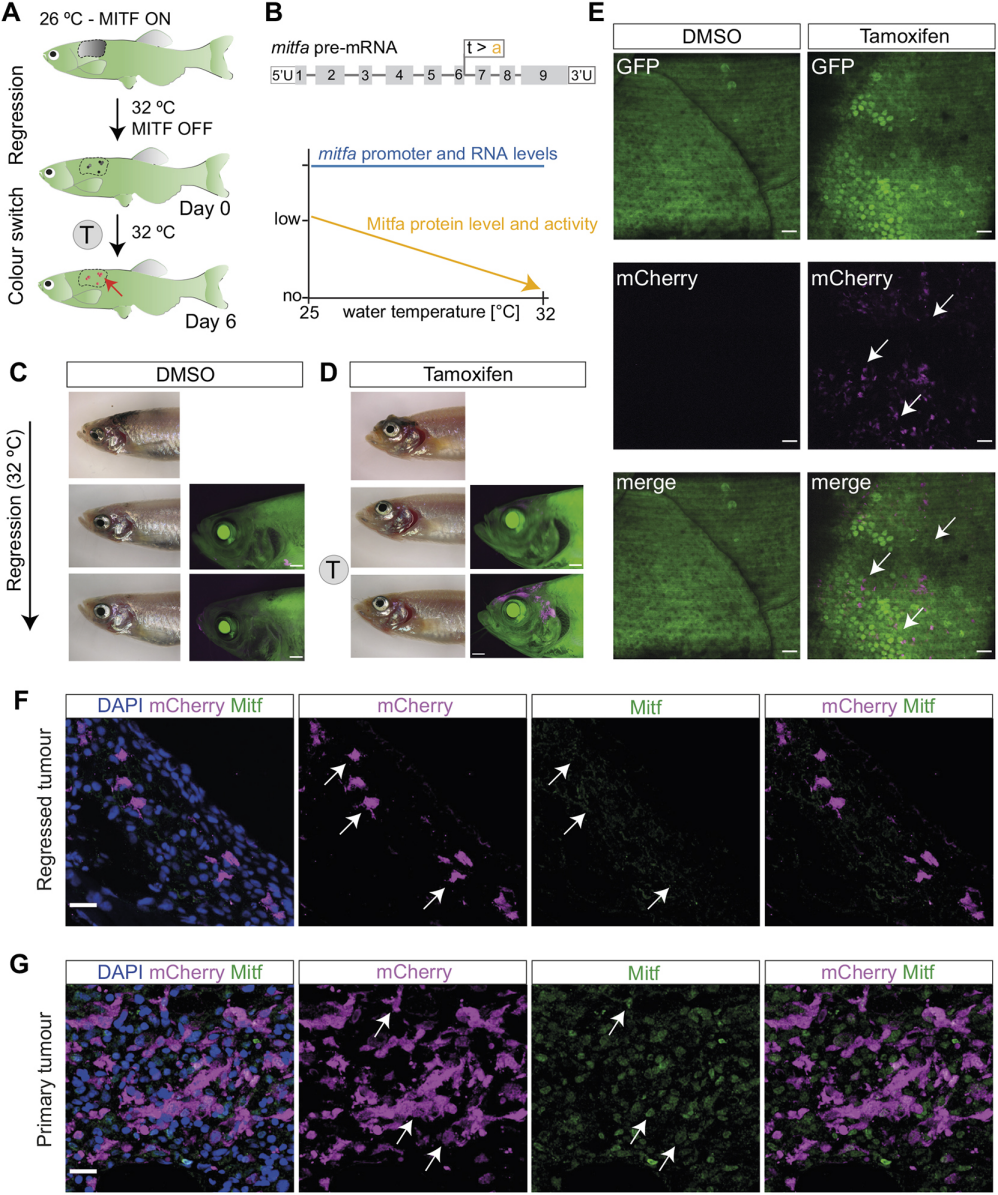
**Successful GFP-to-mCherry fluorophore switch in persister cells.** (A) Schematic representation of the experimental workflow of tamoxifen (T) treatment following melanoma regression. The red arrow points to the location of recombined melanoma persister cells at MRD site. (B) Overview of *mitfa^vc7^* mutant controlling Mitfa activity. (Top) Schematic of *mitfa* pre-RNA with the location of *vc7* mutation at the end of exon 6. (Bottom) Graphical overview of the effect of *mitfa^vc7^* mutation on Mitfa protein level and activity (orange) at increasing water temperatures. Mitfa protein and activity levels decrease with increasing water temperature, unlike the *mitfa* RNA levels, which remain expressed (blue). Schematic adapted from Travnickova et al. (2019). This image is not published under the terms of the CC-BY license of this article. For permission to reuse, please see Travnickova et al. (2019). (C) A representative image of a control fish with no mCherry^+^ cells detectable at the MRD site without tamoxifen treatment (top). Following melanoma regression and subsequent treatment with DMSO (0.04% at 31-32°C) there is no green-to-red recombination in the melanoma MRD (bottom row) compared to pre-treatment (middle row). Scale bars: 1 mm, *N*=3 fish. (D) A representative image of a tamoxifen-treated fish with newly ‘switched’ mCherry^+^ cells (magenta) detectable at the MRD site (top). Following melanoma regression and subsequent treatment with tamoxifen (4 µM, at 31-32°C) there is specific green-to-red recombination in the melanoma MRD (bottom row) compared to pre-treatment (middle row). Scale bars: 1 mm, *N*=3 fish. (E) STD projections of confocal *z*-stack acquisitions showing MRD sites in fish treated with DMSO or tamoxifen. White arrows indicate clusters of mCherry^+^ cells present only in tamoxifen-treated condition. Scale bars: 50 µm, *N*=3 fish. (F) Melanoma cells at the MRD site express mCherry, but lack Mitfa protein (white arrows). STD projections of confocal *z*-stack acquisitions of immunofluorescence staining of the tamoxifen-treated MRD with antibodies for mCherry and Mitfa proteins and with DAPI nuclear staining. Scale bar: 15 µm. (G) Melanoma cells in the primary tumour express both mCherry and Mitfa protein (white arrows). STD projections of confocal *z*-stack acquisitions of immunofluorescence staining of the tamoxifen-treated primary tumour, showing staining of mCherry and Mitfa proteins with DAPI nuclear staining Scale bar: 15 µm. See also Fig. S2.

Next, we asked if the tamoxifen-induced GFP-to-mCherry switch could also be applied to melanoma persister cells in the MRD site (Fig. 5A). We transferred our melanoma fish to a higher water temperature to prevent the correct splicing of *mitfa* (and to thereby turn off Mitfa protein activity) to cause melanoma regression. Because the *mitfa^vc7^* mutation is an RNA-splicing mutation, the expression of *mitfa* or reporters under the control of *mitfa* (e.g. *mitfa:GFP*; *mitfa:cre^ERt2^*) are not affected at the restrictive temperature (Fig. 5B). Once the melanoma had fully regressed, we treated the fish with tamoxifen. We detected mCherry^+^ cells 6 days after the start of tamoxifen treatment at the regression site (Fig. 5C,D). Using confocal microscopy, we were able to validate the presence of mCherry^+^ cells in the tamoxifen-treated group (Fig. 5E) that were morphologically similar to our previous observations using a *Tg(mitfa:GFP)* transgenic line (Travnickova et al., 2019).

We have previously demonstrated that melanoma persister cells in the MRD site do not express Mitfa protein (called MITF independent), but maintain a neural crest identity and express Sox10 (Travnickova et al., 2019). To evaluate if the ‘switched’ mCherry^+^ cells retain the same properties, we immunostained the primary and regressed tamoxifen-treated melanoma tissue sections with mCherry and Mitfa antibodies (Fig. 5F,G)*.* No Mitfa expression was detected in ‘switched’ mCherry^+^ melanoma cells in the regressed tumour. In contrast, the primary tumour showed abundant Mitfa expression in mCherry ‘switched’ cells (Fig. 5F,G). Consecutive sections of the MRD site were stained with mCherry and Sox10 antibodies and confirmed that the mCherry^+^ cells were Sox10^+^ persister melanoma cells (Fig. S2). These data show that ‘switched’ mCherry^+^ persister cells at the MRD site are MITF independent.

**Fig. 6.**
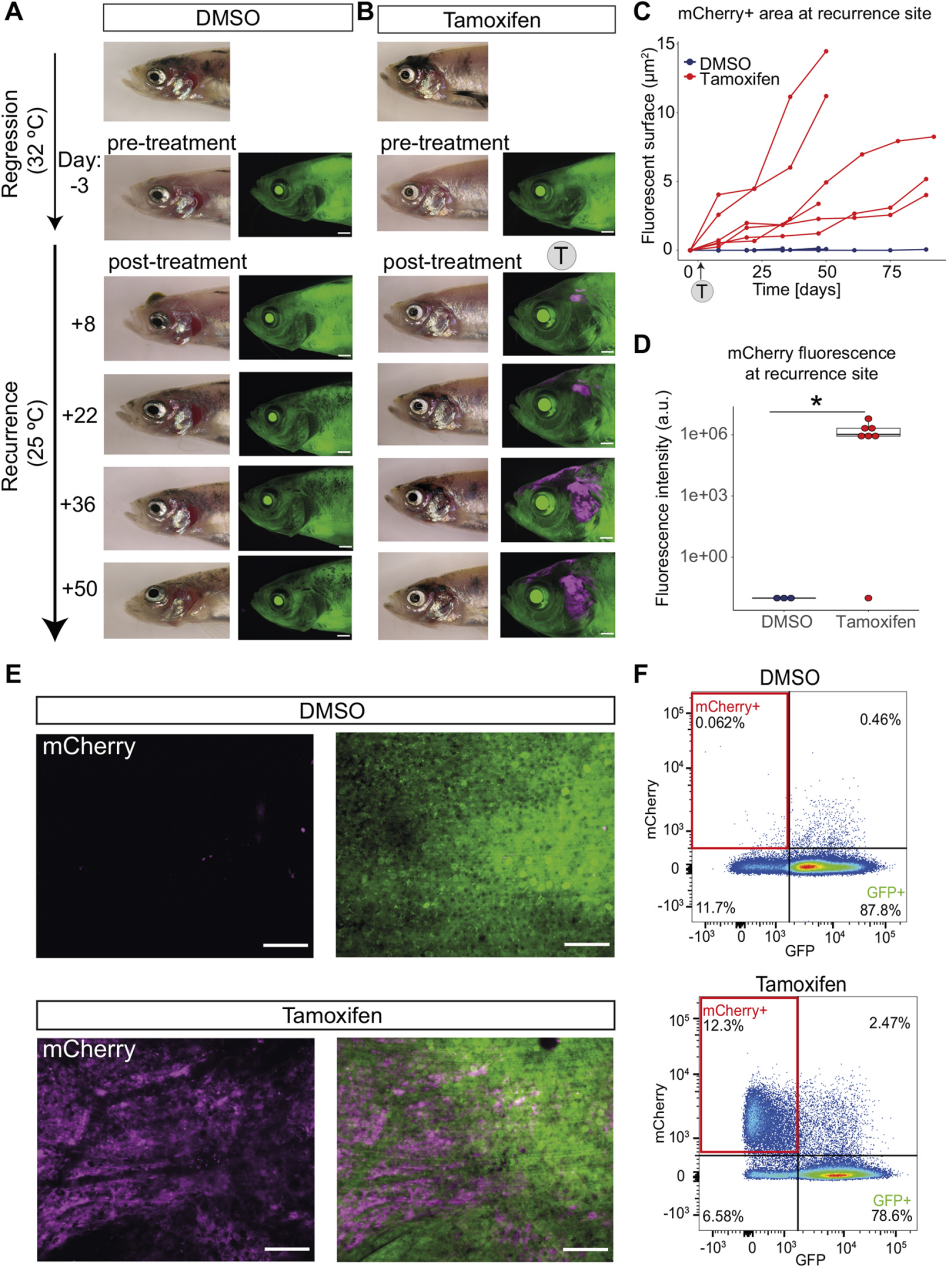
**Persister cells directly contribute to melanoma recurrent disease.** (A) Representative images of control fish with no mCherry fluorescence. A fish with a regressed melanoma (top image, primary tumour; below, regressed tumour) treated with vehicle (0.04-0.05% DMSO at 32°C) showing no green-to-red fluorophore switch in the melanoma MRD compared to the pre-treatment image. Follow-up brightfield and fluorescent acquisitions (every 14 days) show progression of tumour recurrence over time with no appearance or increase in mCherry fluorescence. Scale bars: 1 mm, *N*=5 fish (sum of two independent experiments). (B) mCherry^+^ persister cells from MRD are present through to recurrence. A fish with a regressed melanoma (top image, primary tumour; below, regressed tumour) treated with 4-5 µM tamoxifen (at 32°C) showing green-to-red fluorophore switch (in magenta) in the melanoma MRD. Follow-up brightfield and fluorescent acquisitions (every 14 days) show progression of tumour recurrence over time with increasing mCherry signal. Scale bars: 1 mm, *N*=7 fish (sum of two independent experiments). (C) mCherry^+^ persister cells increase over time. Line plot of area quantification of mCherry signal using fluorescent acquisitions over time during melanoma recurrence. DMSO (blue), *N*=5 fish; tamoxifen-treated group (red), *N*=6 fish (sum of two independent experiments for both groups). Trajectories of melanoma growth represent individual fish. (D) Box plot of fluorescence intensity quantification of mCherry signal of recurrent disease that has grown from MRD treated with tamoxifen. Intensity was measured using average intensity projections of confocal images of recurrence sites comparing DMSO to tamoxifen condition. Lines in boxes indicate the medians, hinges correspond to 25th and 75th percentiles, and whiskers indicate data within 1.5 interquartile range of the upper and lower quartiles. Dots beyond end of whiskers represent outliers. DMSO, *N*=3 fish; tamoxifen-treated group, *N*=4 fish with seven recurred melanomas; *P<0.05, Wilcoxon test. Outlier sample in tamoxifen treatment group is likely to be false negative as mCherry fluorescence was later confirmed using flow cytometry in this highly pigmented sample. a.u., arbitrary units. (E) mCherry is present only in recurrent disease that has grown from MRD treated with tamoxifen. STD projections of *z*-stack confocal acquisition show recurred melanoma sites of fish treated with vehicle (DMSO, top row) or tamoxifen (bottom row) at the MRD stage. Scale bars: 100 µm. (F) mCherry^+^ cells in recurrent disease are detectable by fluorescence-activated cell sorting. A representative pseudocolour flow cytometry plot of a recurred melanoma lesion from DMSO-treated fish and tamoxifen-treated fish. mCherry^+^ cells highlighted in red rectangles (12.3% versus 0.062%). DMSO, *N*= 4 fish; tamoxifen, *N*=5 fish (sum of two independent experiments).

Melanoma persister cells have been proposed to contribute to disease recurrence and drug resistance (Marin-Bejar et al., 2021; Rambow et al., 2018; Shen et al., 2020a; Travnickova et al., 2019; Vendramin et al., 2021), but this has not been demonstrated in an animal model using lineage tracing. Having validated the successful GFP-to-mCherry switch at the MRD site, we next sought to follow the persister cells during recurrence. Once again, we performed tamoxifen treatment and detected GFP-to-mCherry switched persister cells at the MRD site. We then restored MITF activity by lowering the water temperature and followed melanoma recurrence. Over 50 days, the mCherry^+^ cells continuously increased concomitantly with melanoma recurrence (Fig. 6A,B). Fluorescence area quantification of individual fish MRD sites show that mCherry^+^ cells drive tumour growth over time (Fig. 6C). This demonstrates that persister cells directly contribute to the growth of recurrent disease.

Confocal microscopy enabled us to visualise the tumours at cellular resolution, showing fields of mCherry^+^ melanoma cells in recurrent disease (Fig. 6D,E). Quantification of the mCherry^+^ fluorescence indicated that most recurrent disease sites expressed mCherry (Fig. 6D; 6/7 recurrence sites with an average intensity of 2×10^5^ arbitrary units). For one sample, we found that the fluorescence was hindered by strong pigmentation. Given this, we validated the presence of mCherry^+^ cells in this and other recurrent tumours using flow cytometry and could clearly detect mCherry even in highly pigmented samples (Fig. 6F).

### Transcriptional plasticity of melanoma persister cells in recurrent disease

**Fig. 7.**
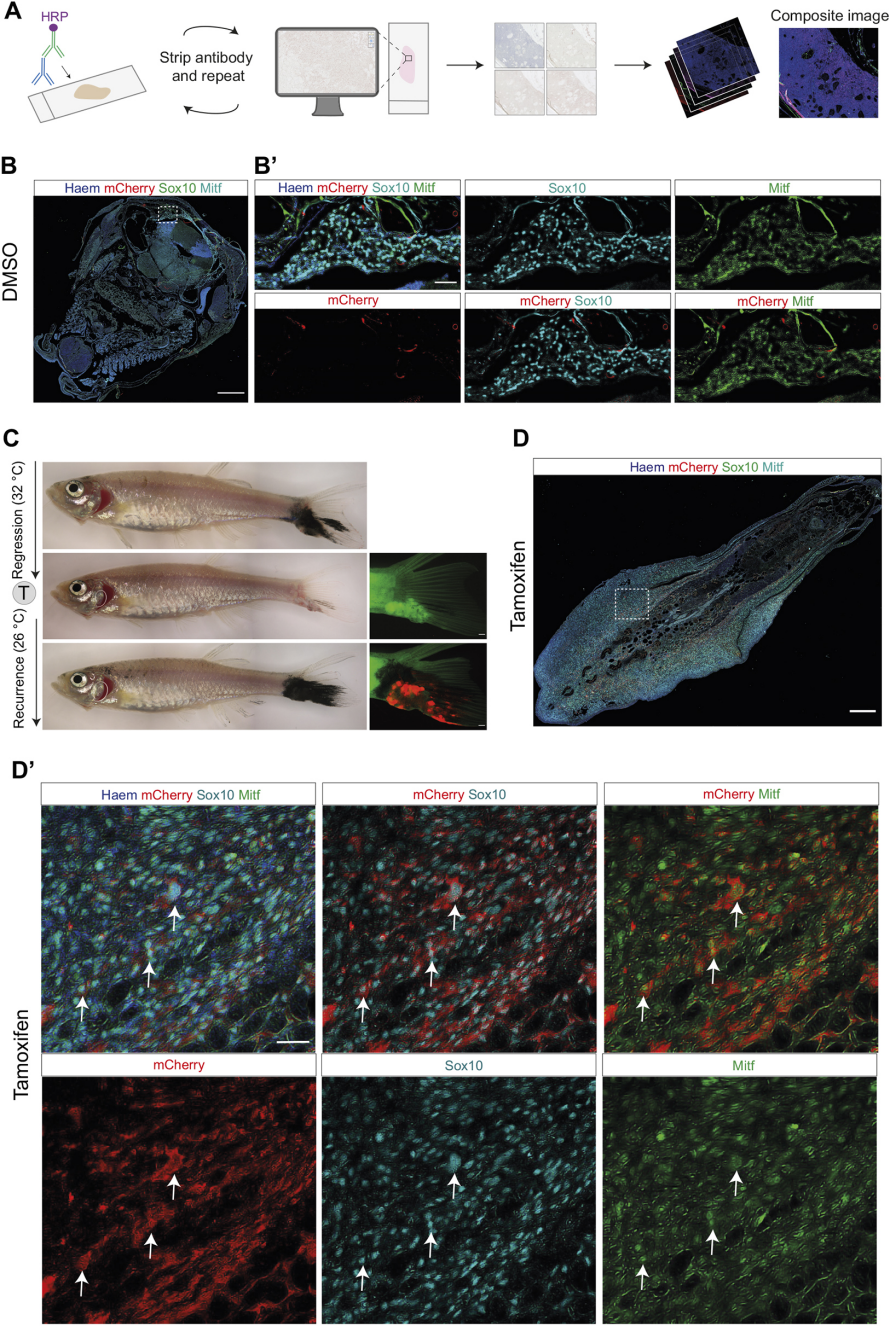
**MITF-independent persister cells regain Mitfa protein expression in recurrent disease.** (A) Overview of the multiplex immunohistochemistry protocol. The antibody staining is repeatedly imaged and stripped, and images are pseudocoloured and stacked on ImageJ to generate a composite image. HRP, horseradish peroxidase. (B) DMSO-treated melanoma recurrent disease on top of the head, labelled with Sox10, mCherry and Mitfa antibodies overlaid with Haematoxylin counterstain; white dashed line box indicates area of section shown in B′. Scale bar: 250 μm. (B′) Enlarged views of DMSO-treated tumour section shown in B, showing lack of mCherry staining in melanoma cells positive for Sox10 and Mitfa. Scale bar: 50 μm. Observable mCherry signal represents trapped antibody in loose tissue fragments. (C) (Left, top to bottom) Brightfield images of melanoma tail fin tumour through growth, regression and recurrence. (Right, middle and bottom) Fluorescent images of regressed and recurred melanoma tail fin tumour, showing mCherry fluorescence in the recurred tumour following tamoxifen-mediated recombination in the MRD. Scale bars: 500 μm. (D) Recurrent disease from MITF-independent melanoma persister cells regain Mitfa protein expression. Overview of tamoxifen-treated melanoma recurrent disease in the tail fin, showing Sox10, mCherry and Mitfa protein staining overlaid with Haematoxylin counterstain; white dashed line box indicates area of section shown in D′. Scale bar: 250 μm. (D′) Enlarged views of tumour section shown in D, showing individual staining of Sox10, Mitfa and mCherry proteins. Scale bar: 25 μm. White arrows highlight cells positive for Sox10, Mitfa and mCherry staining. See also Fig. S3.

We hypothesised that persister cells directly lead to recurrent disease by transitioning from an MITF-independent to a Mitfa^+^ state. As previously described and shown here (Fig. 5F,G), persister cells do not express Mitfa protein, but maintain their expression of Sox10 (Travnickova et al., 2019). We applied multiplex immunohistochemistry (MIHC) on sections of the recurred tumours to determine the protein expression levels of Mitfa and other melanoma markers within recurrent disease (Fig. 7A-D). MIHC allows sequential staining and stripping of several antibodies on the same slide (Pirici et al., 2009). We used an antibody against the mCherry protein to label the ‘switched’ cells in tamoxifen-treated fish compared to DMSO control, together with antibodies against the melanoma markers Sox10 and Mitfa (Fig. 7B-D). As anticipated, recurred tumours from DMSO controls lacked mCherry signal in Sox10^+^ and Mitfa^+^ tumour cells (Fig. 7B,B′). By overlaying the images of mCherry staining with Sox10 and Mitfa staining, we could demonstrate that the mCherry^+^ cells within the tamoxifen-treated recurred tumour expressed both Sox10 and Mitfa (Fig. 7D′; Fig. S3). These results indicate that melanoma persister cells undergo a cell state switch from MITF-independent to Mitfa-expressing cells, regaining features of the primary tumour and providing direct evidence of tumour cell transcriptional plasticity *in vivo*.

## DISCUSSION

Transcriptional heterogeneity underlies the high levels of tumour cell plasticity in melanoma. Here, we apply fate mapping and MIHC to tumour cell populations through melanoma disease states to show that (1) persister cells originate from the primary tumour; (2) persister cells directly contribute to melanoma recurrence; and (3) persister cells exhibit plasticity from a MITF-independent state to Mitfa-expressing melanoma cells in recurrent disease. Thus, persister cells will be a critical drug target for delaying (or even preventing) melanoma recurrent disease.

Our system offers the opportunity to understand how individual cell states, and indeed even individual cells, contribute to drug resistance and tumour progression in zebrafish cancer models. Lessons from mouse models indicate that the efficiency of recombination is largely dependent on the expression level and pattern of the chosen promoter, and thus the tamoxifen protocol often needs to be adapted for each *cre^ERt2^* line (Ellisor and Zervas, 2010; Jahn et al., 2018). In particular, the Bally-Cuif group recently established a 4-OHT protocol for mosaic recombination using short-term drug exposure and long-term multiple-day immersion for maximal recombination, similar to our approach (Than-Trong et al., 2020). Hence, as inducible lineage-tracing methods become more widely applied in zebrafish adults, the zebrafish community will benefit from ensuring that tamoxifen protocols for each promoter are easily accessible.

Here, we showed the fate mapping of the *mitfa:cre^ERt2^*-expressing melanoma cell population through disease stages, which represents a major portion of melanoma cells in our model. MITF is one of the universal diagnostic markers of cutaneous melanoma based on its expression in most melanoma cells (Compton et al., 2015). Previously, we and others have demonstrated the level and importance of transcriptional heterogeneity not only in the primary tumour but also in the persister cells at the MRD site (Marin-Bejar et al., 2021; Rambow et al., 2018; Shen et al., 2020b; Travnickova et al., 2019; Wouters et al., 2020). The persister cell states in our zebrafish models share conserved mechanisms with human melanoma cells depleted for MITF or following BRAF inhibitor drug treatment (Dilshat et al., 2021; Rambow et al., 2018; Travnickova et al., 2019). The lineage-tracing system we present here has the potential to enable fate mapping of different transcriptional cell states at the MRD site and evaluate their contribution to disease progression and response to drug treatment (Lu and Patton, 2022). This can be achieved using cell state-specific markers to recombine a subpopulation of the tumour at the desired stage.

In conclusion, we have successfully applied and validated an inducible *cre^ERt2^/loxP* lineage-tracing system in an adult zebrafish melanoma model to follow cell states through disease stages. Combining fate mapping with live imaging, immunohistochemistry (IHC) and MIHC, we provide direct evidence for the contribution of cells at the MRD site to tumour recurrence, whilst simultaneously demonstrating their MITF-independent to Mitfa^+^ cell state shift. This system has the potential for widespread usage in the study of cancer cell plasticity over time and in the evolution of therapy resistance.

## MATERIALS AND METHODS

### Experimental models and husbandry

Zebrafish were maintained in accordance with UK Home Office regulations, UK Animals (Scientific Procedures) Act 1986, amended in 2013, and European Directive 2010/63/EU under project license 70/8000 and P8F7F7E52. All experiments were approved by the Home Office and Animal Welfare and Ethical Review Body (AWERB; University of Edinburgh Ethics Committee).

Fish stocks used were as follows: *mitfa^vc7^* (Johnson et al., 2011; Zeng et al., 2015), *Tg(mitfa-BRAF^V600E^)*, *tp53^M214K^(lf)* (Patton et al., 2005), *Tg(ubb:loxP-EGFP-loxP-mCherry)* (referred to as *ubi:Switch*) (Mosimann et al., 2011), *Tg(ubb:lox2272-loxP-Tomato-lox2272-Cerulean-loxP-YFP)* [referred to as *Tg(ubb:lox2272-loxP-RFP-lox2270-CFP-loxP-YFP)* or *ubi:zebrabow*] (Pan et al., 2013), *Tg(mitfa:cre^ERt2^)* (this study). Combined transgenic and mutant lines were generated by crossing. Adult fish were maintained either at 28.5°C, 25°C or 32°C under 14:10 h light:dark cycles.

### Generation of zebrafish transgenic line *mitfa:cre^ERt2^*

Cre^ERt2^ was amplified by PCR using *pCGA_cre^ERt2^* (Addgene plasmid #14797) as template. A nuclear localisation signal was added to its N-terminal. The gateway primer sequences were *cre^ERt2^gateF* 5′-GGGGACAAGTTTGTACAAAAAAGCAGGCTGCCACCATGCCCAAGAAGAAGAGGAAGGTGTCCAATTTACTGACCGTACACC-3′ and *cre^ERt2^gateR*: 5′-GGGGACCACTTTGTACAAGAAAGCTGGGTTCAAGCTGTGGCAGGGAAAC-3′. The PCR product was cloned into *pDONOR221*, resulting in *pME-cre^ERt2^*, a middle entry vector. Cre^ERt2^ was then cloned together with the 2.1 kb zebrafish *mitfa* promoter into *pDestTol2CG2* destination vector by the Tol2kit gateway cloning method (Kwan et al., 2007), resulting in the *pEXPmitfa-cre^ERt2^* expression vector. For selection purposes, the construct contains an additional GFP-coding sequence expressed from the heart-specific *cmlc2* (*myl7*) promoter. Two nanolitres of mixed *pEXPmitfa-cre^ERt2^* plasmid and *Tol2* mRNA (25 ng µl^−1^ and 35 ng µl^−1^, respectively) were injected into one-cell-stage zebrafish embryos. Zebrafish embryos with the GFP transgenic marker in the heart were selected and grown to adulthood, then bred with wild-type fish to establish stable lines.

### Genotyping

Zebrafish were genotyped using DNA extracted from fin-clipped tissue using DirectPCR lysis reagent (Viagen) complemented with 0.1 mg ml^−1^ proteinase K. Polymerase chain reaction (PCR) was used to establish the mutant allele status *p53^M214K^* and to verify the presence of transgene *mitfa*-*BRAF^V600E^*, as described in detail before (Wojciechowska et al., 2016).

### Temperature-controlled melanoma regression and recurrence

To induce melanoma regression, selected adult melanoma-prone fish homozygous for *mitfa^vc7^* mutation were transferred to tanks with heaters that kept the water temperature at 32°C compared to standard system temperature of 28.5°C. Fish were monitored twice a week for tumour changes and imaged every 2 weeks to visually compare the regression over time. A tumour was considered fully regressed once no melanoma was visually detectable and stayed unchanged during two consecutive time points of monitoring (usual range of regression being 5-10 weeks). To allow melanoma recurrence, regressed fish were transferred to tanks at room temperature (24-26°C), while monitored daily for tumour progression and health condition of the fish. Fish were always imaged prior to transfer from one temperature to another, then imaged regularly to track their progression and monitored daily. Each fish was followed through recurrence until the time point the tumour reached a similar size to the primary tumour, or until reaching the humane end point criteria based on general fish fitness, swimming behaviour and location of tumour (with a maximum of 3 months of recurrent disease growth).

The emergence of melanoma ranged between 2 and 14 months of age. All fish admitted to the experiment were over 3 months old, and all experiments were finished before fish reached 18 months of age. Both females and males were included in the experiments; on average, across experiments, the ratio was 58% females (50-66%) and 42% males (33-50%).

### Tamoxifen and 4-OHT treatment

The tamoxifen treatment protocol was adapted from Gemberling et al. (2015) and Pinzon-Olejua et al. (2017). The tamoxifen stock solution (Cayman Chemicals or Fisher Scientific) was prepared by dissolving tamoxifen powder in DMSO to reach 10 mM concentration. We prepared fresh stock for each round of treatment on day 0 for the whole 3 day treatment course and stored it at −20°C in 200-250 µl aliquots. Tamoxifen stock solution (or corresponding DMSO volume as a control) was diluted with 500 ml E3 medium (5 mM NaCl, 0.17 mM KCl, 0.33 mM CaCl_2_, 0.33 mM MgSO_4_) in a fish carrier tank, and fish were immersed in the solution for 11-12 h overnight (protected from light using a black chamber box or by wrapping the tank in aluminium foil) for three consecutive nights, with a drug pause during the day. For the tamoxifen treatment of fish with regressed melanoma, the carrier tanks with fish immersed in the tamoxifen or DMSO were kept in a water bath set at 31°C to ensure the continuous suppression of MITF activity.

4-OHT (Sigma, H6278) stock was prepared by dissolving the powder in ethanol to reach 5 or 10 mM concentration and thoroughly vortexed and aliquoted into 100 µl aliquots. The 4-OHT stock solution was then diluted into 200 µM intermediate stock in E3, vortexed thoroughly while protected from light and then diluted in E3 to the final working concentration, 20 µM. Embryos were placed into six-well plates with 3 ml 20 µM 4-OHT, protected from the light and incubated at 28°C. The 4-OHT was refreshed daily for a total incubation of 3 days. Embryos were then washed three times with E3 and incubated in E3 prior to imaging. In Fig. 1D,E, embryos were incubated for 6 h after the end of 4-OHT treatment.

### Live imaging of adult zebrafish

Fish were briefly anaesthetised in tricaine solution (0.1 g l^−1^ MS-222), placed onto a Petri dish while immersed in the tricaine solution and imaged using a Nikon COOLPIX5400 camera attached to a brightfield microscope (Nikon SMZ1500). Each fish was imaged using three consecutive photo shots that were later merged together using Adobe Photoshop automerge function. For fluorescent imaging, anaesthetised fish were imaged under a Leica MZFIII stereomicroscope equipped with a Retiga R1 camera operated via µManager software (Edelstein et al., 2010). Images were taken using GFP and mCherry fluorescent filters. The fluorescent images were processed using Fiji software version 2.1.0 (Schindelin et al., 2012) for pseudocolouring and adjusting minimal and maximal intensity.

For *zebrabow* live imaging, anaesthetised fish were imaged under a Leica M205FCA stereoscope equipped with Leica K8 camera and operated via LASX software (Leica). Images were taken with a 0.63× objective at 1× or 2.5× zoom using CFP, YFP and mCherry fluorescent filters and processed as above. As shown in Fig. S1B, the CFP channel showed a certain level of autofluorescence, most prominent in the creases of jaw and gills, that was not considered as positive signal.

### Zebrafish tissue and embryo confocal imaging

For confocal microscopy, zebrafish embryos were anaesthetised with 0.1 g l^−1^ MS-222, and adult fish were culled by overdose of anaesthetic (1 g l^−1^ MS-222, followed by death confirmation) and embedded in 1% low-melting-point (LMP) agarose (Invitrogen) with the tumour or regressed tissue oriented to the bottom of six-well glass-bottom plates (Cellvis). For dorsal imaging of zebrafish embryos, fish were incubated for 5 min with 5 mg ml^−1^ adrenaline (epinephrine, Sigma, E4642) in order to contract the pigment in melanocytes prior to mounting in LMP agarose for imaging. Images of *ubi:Switch* transgenic were obtained under an Andor Dragonfly spinning disk confocal microscope equipped with an Andor Zyla sCMOS camera through a 20× air objective with 2048×2048 pixel resolution and 1 µm interval. Images of *ubi:zebrabow* transgenic were obtained under a Leica STELLARIS 8 confocal microscope equipped with white-light laser through 10× and 20× air objectives with 1024×1024 pixel resolution and 1 µm (20×) or 3 µm (10×) intervals. In contrast to stereoscope imaging, we did not detect CFP channel autofluorescence using confocal microscopy.

### Image processing and fluorescence quantifications

Confocal acquisitions were processed using Fiji software version 2.1.0 (Schindelin et al., 2012). Standard deviation intensity projections of all *z*-slices were used except for fluorescence intensity quantification, where average intensity projections were used. Fluorescence intensity has been measured as described (McCloy et al., 2014). Briefly, average intensity projections of *z*-stacks acquired using the Andor Dragonfly spinning disk confocal microscope were split by channel to select mCherry only. Intensity of the whole imaged region was then measured, and intensity of the small region without any tissue was used to determine background. To calculate fluorescence intensity, corrected total cell fluorescence was calculated by subtracting the background fluorescence intensity. Any negative value was replaced by 0. Fluorescence area over time was measured on the mCherry channel of Leica MZFIII epifluorescence microscope acquisitions taken before and after the tamoxifen treatment and then every 2 weeks through melanoma recurrence. Area was manually depicted using the polygon selection tool in Fiji and then measure in µm^2^. Any sample that showed fluorescence signal prior to the tamoxifen treatment was excluded from the analysis. Graphs summarising the fluorescence intensity quantification were made using R software version 4.0.3 via R studio interface version 1.1.456 equipped with ggplot2 package (https://www.r-project.org/; https://www.rstudio.com/; https://ggplot2.tidyverse.org/).

### Vibratome sectioning

Fish were fixed overnight in 4% PFA at 4°C with agitation. The piece of fish tissue was then rinsed twice with PBS for several minutes and mounted in 4% agarose/PBS using a well from a six-well plate as a mould. Any excess agarose above 0.5 cm from the tissue was discarded prior to sectioning. Tissue was sectioned transversely using a Leica VT1200S vibratome at a thickness of 400 µm, and the speed of vibrating blade was 0.1 mm s^−1^. Sections were collected in PBS and mounted in 1% LMP agarose in a six-well glass-bottom plate prior to imaging using the Leica STELLARIS 8 as described above.

### Fluorescence-activated cell sorting

Adult tamoxifen-treated or DMSO control fish with recurred tumours were culled using overdose of tricaine (1 g l^−1^ MS-222, followed by death confirmation). Tumours were individually dissected and chopped using sterile No. 9 blade scalpels. Samples were then dissociated with 0.25 mg ml^−1^ liberase TL (Roche) for 15 min at room temperature while inverting the tube, re-suspended in FACSmax cell dissociation solution (Genlantis) and filtered through a 40 µm cell strainer. Samples were sorted by a FACSAria2 SORP instrument (BD Biosciences UK) equipped with 405 nm, 488 nm and 561 nm lasers. Green fluorescence was detected using GFP 525/50 bandpass (BP) filter and 488 nm laser, red fluorescence was detected using mCherry 610/20 BP filter and 561 nm laser, and live cells were selected as 4′,6-diamidino-2-phenylindole (DAPI) negative using DAPI 450/20 BP filter and 405 nm laser. Data were analysed using FlowJo software (BD Biosciences) version 10.8.1.

### Histology

Fish samples were collected, fixed and processed as described in detail in Wojciechowska et al. (2016). Briefly, tissue was fixed by immersion in 4% PFA at 4°C with agitation for 3 days, decalcified in 0.5 M EDTA (pH 8) at 4°C with agitation for 5 days and then stored in 70% ethanol at 4°C. To obtain sections for pathology analysis, tissue was processed in 95% ethanol, absolute alcohol, xylene and paraffin, embedded in blocks, cut into 5 μm-thick sections and placed onto glass slides. Haematoxylin and Eosin staining and IHC were performed as described in detail in Wojciechowska et al. (2016). The slides were imaged using a Hamamatsu NanoZoomer SlideScanner, and the images were processed using NDP.3 software.

### Immunofluorescence staining

PFA-fixed paraffin-embedded (FFPE) sections were deparaffinised in xylene and gradually rehydrated through baths of ethanol of decreasing concentrations. Sections were bleached in 1% KOH/3% H_2_O_2_ solution for 15 min prior to being subjected to heat-mediated antigen retrieval in citrate buffer (0.01 M, pH 6). Sections were blocked in 10% goat serum for 1 h and incubated with the following primary antibodies: anti-mCherry (Abcam, ab125096, 1:3000 or 1:4000) (Kobayashi et al., 2014), anti-Mitfa (Sigma, SAB2702433, 1:1000) and anti-Sox10 (Abcam, ab125096, 1:4000) (Travnickova et al., 2019) overnight at 4°C. After PBS/0.1% Tween 20 washes, sections were incubated with Alexa Fluor 488 (#A11001) or 546 (#A11003) goat anti-mouse and/or Alexa Fluor 647 (#A31573) goat anti-rabbit secondary antibodies at 1:500 dilution (Invitrogen) and DAPI (Sigma, 1:500) for 30 min in the dark at room temperature and mounted in hydrophilic mounting medium prior to imaging. Sections were imaged using an Andor Dragonfly confocal microscope equipped with Andor Zyla sCMOS camera using 20× and 40× air objectives.

### MIHC

FFPE sections were deparaffinised with xylene before gradual rehydration and bleached in a 1% KOH/3% H_2_O_2_ solution for 15 min. Slides were then stained with Haematoxylin, mounted in hydrophilic mounting medium and imaged using a Hamamatsu NanoZoomer slide scanner. After coverslip removal, slides were subjected to heat-mediated antigen retrieval in citrate buffer (0.01 M, pH 6) for 7 min. The sections were then incubated in serum-free protein blocking solution (DAKO) for 30 min at room temperature and incubated in primary antibody overnight at 4°C. Following TBS/0.1% Tween 20 washes, the sections were incubated in horseradish peroxidase (HRP)-conjugated rabbit/mouse secondary antibody (DAKO Real EnVision kit) for 30 min at room temperature. Antibody staining was revealed via incubation in AEC chromogen (Abcam) for 5-30 min. Following each antibody revealing, the slides were again mounted in hydrophilic mounting medium and imaged using a Hamamatsu NanoZoomer slide scanner. The slides were then de-coverslipped and underwent chromogenic de-staining in an alcohol gradient and subsequent antibody stripping via a 75 min incubation in a solution glycine-SDS, pH 2 at 50°C, before the next blocking and antibody round (adapted from Pirici et al., 2009; Tsujikawa et al., 2017). The following antibodies were used for MIHC: anti-Sox10 (Abcam, ab229331, 1:4000), anti-mCherry (Abcam, ab125096, 1:4000) and anti-Mitfa (Sigma, SAB2702433, 1:1000).

### Statistical analysis

Sample distribution was evaluated using Shapiro–Wilk test. As the samples exhibited non-Gaussian distribution, statistical evaluation was performed using nonparametric Wilcoxon test. We set up the threshold to *P*=0.05 to be considered as statistically significant with the following symbols: **P<0.01; *P<0.05. Statistical analysis was performed using R software version 4.0.3 through R studio interface version 1.1.456 (https://www.r-project.org/; https://www.rstudio.com/).

## Supplementary Material

Supplementary information
